# Modified Potentiometric Screen-Printed Electrodes Based on Imprinting Character for Sodium Deoxycholate Determination

**DOI:** 10.3390/biom10020251

**Published:** 2020-02-06

**Authors:** Ayman H. Kamel, Samar Ezzat, Mona A. Ahmed, Abd El-Galil E. Amr, Abdulrahman A. Almehizia, Mohamed A. Al-Omar

**Affiliations:** 1 Department of Chemistry, Faculty of Science, Ain Shams University, Cairo 11566, Egypt; s.ezzat.ouda@gmail.com; 2 Chemistry Department, College for Women, Ain Shams University, Heliopolis, Cairo 11751, Egypt; abdelaziz.mona@gmail.com; 3 Pharmaceutical Chemistry Department, Drug Exploration & Development Chair (DEDC), College of Pharmacy, King Saud University, Riyadh 11451, Saudi Arabia; mehizia@ksu.edu.sa (A.A.A.); malomar1@ksu.edu.sa (M.A.A.-O.); 4 Applied Organic Chemistry Department, National Research Center, Giza 12622, Egypt

**Keywords:** sodium deoxycholate (NaDC), molecular imprinted polymer (MIP), screen-printed ion selective electrodes, single-walled carbon nanotubes (SWCNTs), human serum albumin (HSA)

## Abstract

Potentiometric sensors have a great influence on the determination of most various compounds in their matrices. Therefore, efficient and new sensors were introduced to measure sodium Deoxycholate (NaDC) as a bile acid salt. These sensors are based on NaDC imprinted polymer (MIP) as sensory element. The MIP beads were synthesized using thermal polymerization pathway, in which acrylamide (AAm), ethylene glycol dimethacrylate (EGDMA), NaDC, and benzoyl peroxide (BPO) were used as the functional monomer, cross-linker, template, and initiator, respectively. The proposed sensors were fabricated using a coated screen-printed platform and the sensing membrane was modified by single-walled carbon nanotubes (SWCNTs) as an ion-to-electron transducer. The sensors exhibited high sensitivity that reached 4.7 × 10^−5^ M of near-Nernestian slope (−60.1 ± 0.9 mV/decade, *r^2^* = 0.999 (*n*= 5)). In addition, the sensors revealed high selectivity, long lifetime, high potential stability, and conductivity that ensure reproducible and accurate results over a long time. MIP characterization was performed using Fourier Transform-Infrared (FT-IR) and a scanning electron microscope (SEM). Regarding the interaction of NaDC with serum albumin (SA), albumin is determined in human serum samples as human serum albumin (HSA), which was collected from different volunteers of different ages and gender.

## 1. Introduction

**Figure 1:**
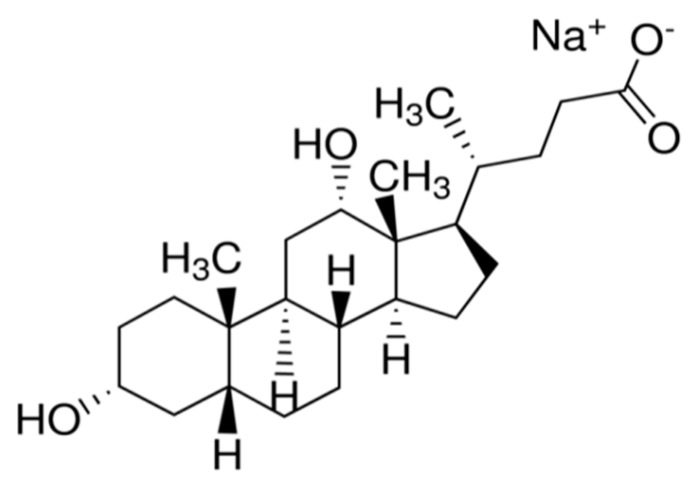
Structure of Sodium deoxycholate (NaDC).

Sodium deoxycholate (NaDC) compound (Figure 1) is considered as one of the bile acid salts that biosynthesized in the liver of the human body from cholesterol moiety. These bile salts have a great biological role as an emulsifiers for different biological compounds in the body such as fat-soluble vitamins in the intestine, bilirubin, cholesterol, and lecithin [1,2]. NaDC has the steroidal skeleton with four rings containing hydroxyl groups and terminating in a carboxylic acid group. It is a water-soluble compound that has a good acceptability for pharmaceutical products and enhances the permeation of different drugs through the biological membranes [3]. In addition, its unique structure, chemical properties, and its high affinity to form protein–NaDC complexes have a crucial impact as bio-surfactant interactions in the determination of the proteins such as serum albumin, which has an important applications in biosciences, foods, cosmetics, and drug delivery [4].

Therefore, there are different methods for the determination and monitoring of bile acids or their protein complexes such as complex NMR spectroscopy [5], steady-state fluorescence, time–resolved fluorescence and fluorescence measurements [1], and volumetric and compressibility studies for bile salts in aqueous solutions [6,7] using viscosity, density, and speed of sound techniques at different temperatures [8]. These previously reported methods of sodium deoxycholate determination as a free compound or its complexes have clear disadvantages that can be shown in the preliminary steps before the detection and monitoring, including time consumption, hard work, and complicated theories of detection. Therefore, the potentiometric methods based on ion-selective electrodes (ISEs) of screen-printed solid contact type [9,10] can be utilized due to their accessibility, high sensitivity, miniaturization, simplicity, and low cost features in the monitoring process. 

ISEs are one of the most important analytical methods due to their ability for enhancement of the detection efficiency with different ionophores, which have specific characteristics and features capable of simple, selective, and sensitive determination of different analytes in aqueous and biological solutions [11,12,13]. Among these ionophores, there are molecular imprinted polymers (MIPs), which can be manufactured with specific characters that compatible with their detection of the analyte in the different solutions. MIPs are synthesized with the thermal polymerization of functional monomer with cross-linker and template in the presence of initiator and appropriate progen. These polymers formed as an intermediate complex with unique features can be left after washing with suitable solvent [14,15]. Potentiometric sensors based on molecularly imprinted polymers (MIPs) are now exhibiting a great potential to significantly change the view of using non-affordable ionophores. These ionophores are characterized by their high cost, or using ion exchangers, and characterized by their poor selectivity. On the other hand, MIPs are thermally stable, costly effective, and easier to synthesize [16,17]. Currently, potentiometric sensors based on different MIP receptors have been synthesized for determination of different organic species in either their ionic or neutral forms [16,18,19,20]. Few reports are presented in the literature for imprinted synthetic receptors for deoxycholate [21,22].

Herein, we present for the first time a novel potentiometric method for the assessment of deoxycholate sodium as a bile acid conjugate. The method is based on the use of solid contact screen-printed ceramic chip modified with SWCNT as a conductive material. MIP was integrated with the sensor as a recognition ionophore and dispersed in a plasticized polyvinyl chloride (PVC) membrane. The different features of the proposed sensor were studied such as sensitivity, selectivity, pH effect, and potential stability. The electrochemical measurements such as electrochemical impedance spectroscopy (EIS) and chronopotentiometric (CP) measurements were used to evaluate the capacitative character of SWCNTs as an ion-to-electron transducer. Based on the interaction of NaDC with different proteins such as human serum albumin (HSA), albumin is kinetically determined in different human serum samples using the proposed electrode. The results were compared with the standard reference method for determining HSA.

## 2. Materials and Methods

### 2.1. Apparatus

MIP beads were characterized using Fourier-transform infrared spectroscopy (FT-IR) spectra that were investigated using modified FT-IR spectrometer with attenuated total reflection (ATR) (Alpha II, Bruker ABCO, Cairo, Egypt). Furthermore, scanning electron microscopy (SEM) was used for showing surface morphology (JOEL, Osaka, Japan). All the potentiometric measurements were carried out using Orion (Cambridge, MA, USA) Model 720 /SA pH /mV meter accompanied with Orion Ag/AgCl double-junction reference electrode (Model 90-20) filled with 10% (*w*/*v*) KNO_3_ and NaDC/ISE at ambient temperature. Chronopotentiometry (CP) and electrochemical impedance measurements (EIS) of the proposed sensor based MIP were evaluated by applying constant-current using a one-compartment three-electrode cell by utilizing an Metrohom potentiostat/galvanostat (Autolab, model 204, NOVA 1.1 software; Metrohm Auto lab B.V. Utrecht, The Netherlands).

### 2.2. Reagents and Materials

Sodium deoxycholate (NaDC), acrylamide monomer (AAm), ethylene glycol dimethacrylate cross- linker (EGDMA), benzoyl peroxide (BPO), Human serum albumin (HSA) with a molecular weight (69 kDa) and absolute ethanol were obtained from Sigma-Aldrich (St. Louis, MO, USA). High molecular weight poly (vinyl chloride) (PVC), dioctyl phthalate plasticizer (DOP), aliquate 336S and tetrahydrofuran (THF) were obtained from Fluka AG (Buchs, Switzerland). Several aqueous solutions of NaDC were prepared with freshly de-ionized water (18.2 MΩ cm specific resistance). The appropriate buffer for the potentiometric measurements was prepared from 30 mM NaHCO_3_-Na_2_CO_3_ buffer, pH 9.2, ionic strength 0.1M NaCl.HSA stock solution (100 mg/dL) was prepared by dissolve 0.1 g of bovine serum albumin and 20 mg of sodium azide in water in a 100-mL volumetric flask. The solution was diluted to the volume with water and stored at 4 °C. Working HSA solutions were prepared by appropriate dilutions of the stock albumin standard solution and stored at 4 °C. Albumin solutions containing 20 mg/dL sodium azide show no evidence of bacterial growth after storage for 6 months at 4 °C.

### 2.3. Molecular Imprinted (MIP) and Non-Imprinted Polymers (NIP) Synthesis

MIP was prepared by thermal polymerization by adding deoxycholic acid-sodium salt (NaDC) as a template; AAm (monomer) and EGDMA (cross-linker) with amounts were valued as 0.5, 3.0 and 3.0 mmol, respectively. All the components were placed in a 25 mL capped glass tube and its mixture was left for 1 h for pre-complex formation. After that, 80 mg of benzoyl peroxide (BPO) and 15 mL of absolute ethanol were added. This mixture was purged with nitrogen gas under a gentle flow for 10 min. The polymerization process was carried for 18 h in an oil bath at 80 °C. The obtained polymer was washed from the undesired species and the template molecule several times with an absolute ethanol in soxhlet for 48 h. The MIP beads were left till complete dryness at ambient temperature before using as an ionophore in sensor membranes. NIP was prepared with the same method in absence of the template. 

### 2.4. Membranes and Electrode Construction

For sensor establishment, the plasticized PVC membrane was prepared by dissolving 30 mg of MIP, 62 mg of PVC, 4 mg of aliquat and 102 mg of DOP in 2 mL of THF. The cocktail was drop casted on the carbon orifice of the screen-printed electrode that previously coated with (10 µL) SWCNTs (5 mg/mL THF) with 10 µL and was left until complete drying. After that, the electrochemical cell was established that consists of the proposed sensor versus the reference electrode (Orion Ag/AgCl) for potentiometric measurements. NIP calibration was measured with the membrane consists of the same amounts of all components, in which MIP was replaced by NIP.

### 2.5. Potentiometric Assay of Human Serum Albumin (HSA)

A calibration plot for albumin was made by transferring 10 mL of 1.0 × 10^−2^ M NaDC, pH 9.2 to a 25-mL beaker, and the NaDC PVC membrane sensor, in conjunction with a double junction Ag/AgCl reference electrode was immersed in the solution. After a constant potential reading was obtained, 100 µL aliquots containing 0.1–1.0 mg/dL of albumin was added. The rate curves (potential/time) were plotted, and the maximum initial rate of potential change expressed as (Δ*E*/Δ*t*) was graphically obtained using the rate portion of the curve. The initial rate was plotted as a function of the albumin concentration. The calibration curve was used for subsequent measurements of unknown albumin concentration. A blank experiment was carried out under similar conditions in absence of albumin.

For assay of human serum albumin (HSA), the above mentioned steps were repeated but using 100 µL aliquot of the serum sample (obtained from volunteers from different ages and genders) instead of the reference albumin solution. The rate curve was recorded, and the maximum initial rate of the potential change expressed as (Δ*E*/Δ*t*) was graphically determined and compared with the calibration plot.

## 3. Results and Discussion

### 3.1. Characterization of MIP Particles

**Figure 2:**
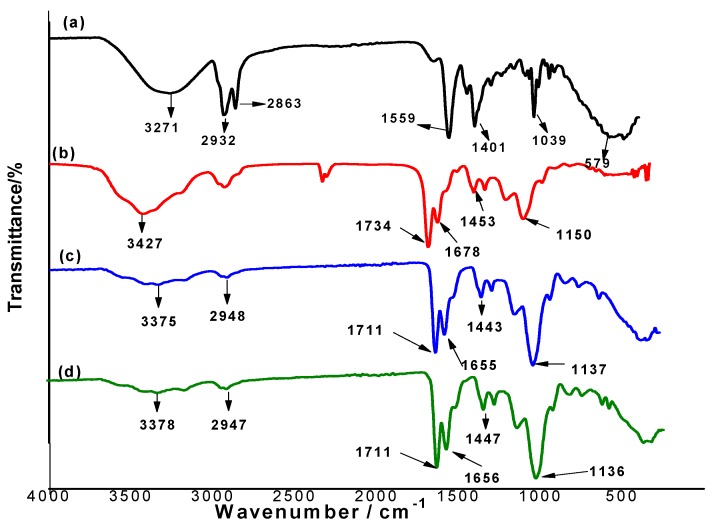
Fourier Transform-Infrared (FT-IR) spectra for (**a**) NaDC, (**b**) NaDC/MIP, (**c**) washed MIP and (**d**) NIP beads.

The FT-IR spectra of sodium deoxycholate, un-washed MIP, washed MIP and NIP are shown in Figure 2a–d. The FT-IR spectrum of sodium deoxycholate (Figure 2a) showed a broad band in the range 3408–3164 cm^−1^ which is assigned for stretching O–H bond. In addition, assignable –C=O group of carboxylate ester group in NaDC is represented as sharp peak at 1559 cm^−1^. The peaks at 2932 and 2863 cm^−1^ are assignable to the stretching C–H bond. Another sharp peak appeared at 1039 cm^−1^ and assigned for CCO bonds. The FT-IR spectra of both washed MIP and NIP are completely similar (Figure 2c,d). They showed sharp peaks at 1717 and 1136–1142 cm^−1^ for stretching –C=O and –C–O, respectively. Those are coming from the EGDMA cross-linker. The peak at 1670 is assigned to the amide carbonyl group coming from the AAm monomer. Broad peaks appeared in both spectra in the range of 3534 to 3229 cm^−1^ revealed to the N–H (stretching) that attend from the AAm monomer.

As shown in Figure 2b, both the peaks of –C=O group of carboxylate ester group and CCO bonds in NaDC was completely diminished. This can be attributed to the contribution of COO– group in NaDC in the complexation with the skeletal structure of the imprinted polymer. In addition, the stretching C=O coming from either EGDMA or AAm are shifted and located at 1734 and 1678 cm^−1^, respectively. This can confirm that these groups have a great contribution towards the complexation with the functional groups in NaDC molecule.

**Figure 3:**
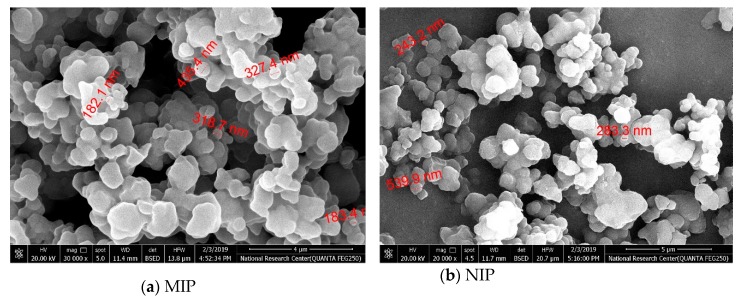
SEM images of (**a**) MIP and (**b**) NIP beads.

The morphologies of both MIP and NIP beads were investigated and presented in the SEM micrographs in Figure 3. As shown in Figure 3a, the imprinted particles were of spherical and semi-uniformed size with a diameter distribution of 180–400 nm. These uniform-sized nano-beads give a good dispersion in the polymeric ISE membrane. This could reduce the membrane resistance and induce more binding sites available in the membrane. The preparation of NIP nano-beads using the same protocol showed a similar morphology as in the MIP beads but with a size ranged between 250–539 nm diameters (Figure 3b).

### 3.2. Membrane Optimization

The potentiometric features of the proposed sensor can be affected with MIP amount that is clarified in Table 1. For ISEs based on MIP only (sensor 2), they revealed an anionic sub-Nernestian slopes (−41.6 ± 1.5) mV/decade with detection limits values of 3.1 × 10^−4^ M. With the addition of Aliquat 336, as a cationic additive (sensor 3), the slope and detection limit were enhanced to be (−47.1 ± 1.8) mV/decade and 2.5 × 10^−4^ M, respectively. Blank membrane-based sensor (sensor 1) that contains only aliquat 336 without MIP revealed a slope of −32.6 ± 0.7 mV/decade and detection limit of 3.5 × 10^−3^ M.

**Figure 4:**
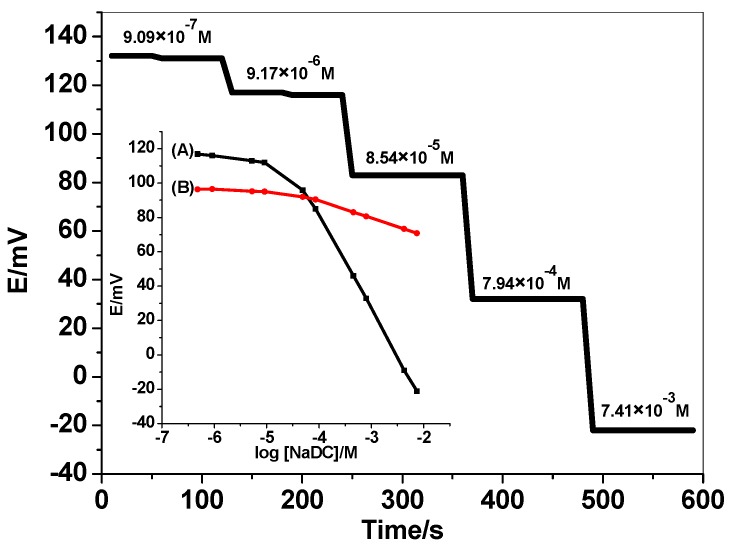
Potentiometric response curves obtained screen-printed ion-selective electrodes (ISEs) integrated with (**A**) MIP and (**B**) NIP.

For more optimization for the testing membrane, the amount of MIP in the membrane sensor was also tested. As mentioned, the slope and detection limit of the proposed sensors are enhanced as the amount of MIP in the plasticized membrane increases from 5 to 30 mg. They begin to decline when 35 mg of MIP is dispersed in the membrane. First, 5, 10, and 20 mg of MIP based sensors revealed anionic sub-Nernestian slopes (−47.1 ± 1.8), (−48.8 ± 2.1), and (−50.3 ± 1.4) mV/decade with detection limits values of 2.5 × 10^−4^, 2.0 × 10^−4^ M, and 7.5 × 10^−5^, respectively. Then, extremely increasing of the MIP amount until reached to 30 mg enhanced the slope to be near-Nernestian slope (−60.1 ± 0.9) of *r^2^* = 0.999 mV/decade and exhibited low detection limit (4.7 × 10^−5^ M) due to its high affinity for NaDC ions in the solution that confirm host-tailored efficiency of MIP-NaDC in the monitoring of NaDC. Further addition of MIP in the membrane (i.e., 35 mg), the slope and detection limit were declined to be −51.2 ± 0.1 and 1.0 × 10^−4^, respectively. Therefore, 30 mg of MIP was chosen as the optimized amount of the MIP and its feature was represented in Figure 4. As a control, membrane sensors based on NIP nano-beads were also tested and shown in Figure 3.

Steady potential response (±1.2 mV) of MIP-NaDC sensor was reached after 10 s using the proposed sensor in 1.0 × 10^−5^–1.0 × 10^−2^ M NaDC solutions with a rapid 10-fold and recording the potential values over 2 min for each concentration. This rapid response of time confirm that the applicability, sensitivity, and efficiency of the proposed sensor in the NaDC monitoring in the solutions.

**Figure 5:**
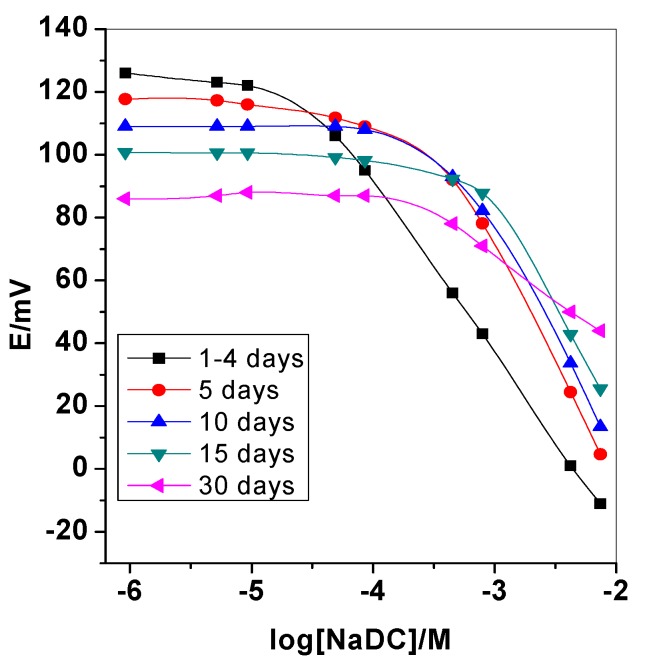
Life-span of the proposed deoxycholate membrane based sensor.

The life span of the proposed sensors was also tested because it is an important feature for the industrial life [23]. Therefore, studying MIP features day-to-day occurred by daily calibration with the proposed sensor. As shown in Figure 5, the calibration slope and detection limit were constants over four working days. From the 5th day to the 15th day, both calibration slope and detection limit begin to decline. After 30 working days, a noticeable electrode failure is observed. So, we can conclude that all sensors examined, the detection limits, response times, linear range and calibration slopes were reproducible within their original values over a period of at least one week. 

**Figure 6:**
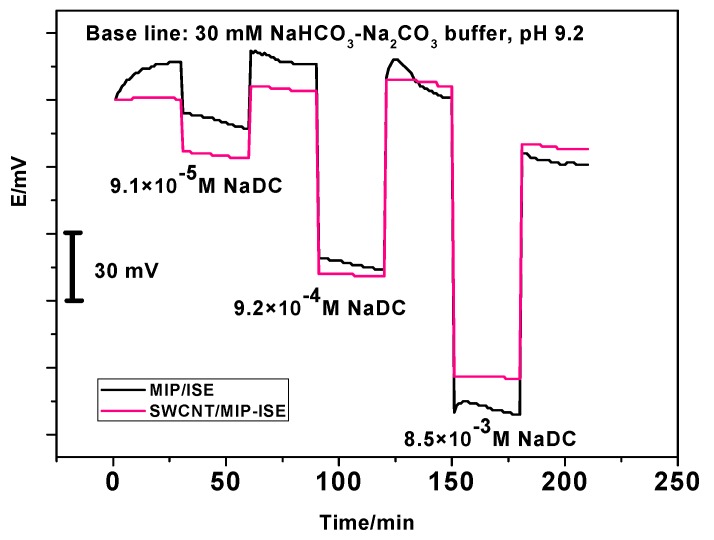
Water-layer tests for NaDC–ISE with and without single-walled carbon nanotubes (SWCNTs) as the solid contact.

To study the lipophilicity of SWCNTs layer and absence of water layer between the sensing membrane and electronic conductor substrate, water layer test was carried out. The test was carried out by measuring the electrode potential at first in 30 mM NaHCO_3_/Na_2_CO_3_ buffer of pH 9.2 for one hour followed by measuring it in different concentrations of NaDC (9.1 × 10^−5^, 9.2 × 10^−4^, a d 8.5 × 10^−3^ M), which separated and determined with 30 mM NaHCO_3_/Na_2_CO_3_ buffer of pH 9.2. As shown in Figure 6, in the case of the absence of SWCNT layer, a potential drift was observed with the value of ~30 mV in 1 h when the electrode was inserted in the buffer. Subsequently, when the solution was changed to 9.1 × 10^−5^ M NaDC solution, a potential drift of ~15 mV occurred for the second hour, while using NaDC concentration (9.2 × 10^−4^ M) a potential drift of ~8 mV occurred. Notably, in the case of the presence of the SWCNT layer, nearly no significant potential drifts (~3 mV) were observed when the measured solution was changed from 9.2 × 10^−4^ M to 8.3 × 10^−3^ M. So, it can be clear that formation of a water layer between the membrane and the screen-printed platform that influenced the potential stability over time. On the other hand, this issue was resolved by adding the SWCNT layer between the membrane and screen-printed chip to prevent water layer formation and enhanced the potential stability.

To study the lipophilicity of SWCNTs layer and absence of water layer between the sensing membrane and electronic conductor substrate, water layer test was carried out. The test was carried out by measuring the electrode potential at first in 30 mM NaHCO_3_/Na_2_CO_3_ buffer of pH 9.2 for one hour followed by measuring it in different concentrations of NaDC (9.1 × 10^−5^, 9.2 × 10^−4^, a d 8.5 × 10^−3^ M), which separated and determined with 30 mM NaHCO_3_/Na_2_CO_3_ buffer of pH 9.2. As shown in Figure 6, in the case of the absence of SWCNT layer, a potential drift was observed with the value of ~30 mV in 1 h when the electrode was inserted in the buffer. Subsequently, when the solution was changed to 9.1 × 10^−5^ M NaDC solution, a potential drift of ~15 mV occurred for the second hour, while using NaDC concentration (9.2 × 10^−4^ M) a potential drift of ~8 mV occurred. Notably, in the case of the presence of the SWCNT layer, nearly no significant potential drifts (~3 mV) were observed when the measured solution was changed from 9.2 × 10^−4^ M to 8.3 × 10^−3^ M. So, it can be clear that formation of a water layer between the membrane and the screen-printed platform that influenced the potential stability over time. On the other hand, this issue was resolved by adding the SWCNT layer between the membrane and screen-printed chip to prevent water layer formation and enhanced the potential stability.

### 3.3. Potential Stability

**Figure 7:**
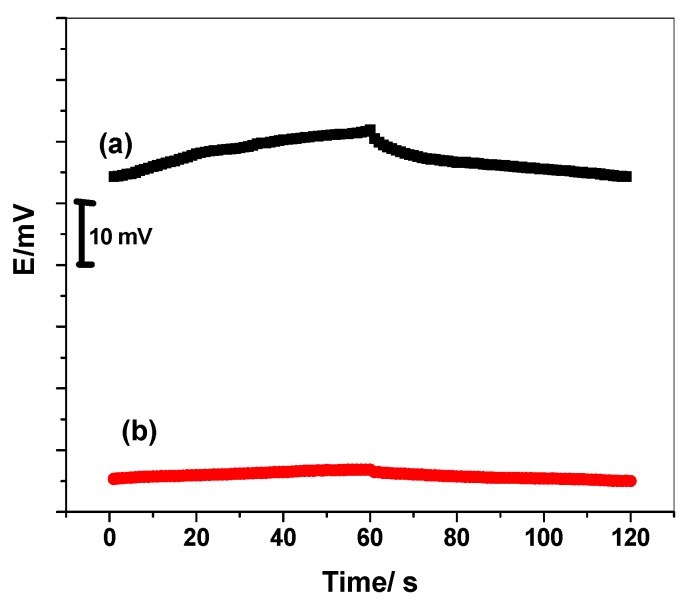
Chronopotentiometry for NaDC/MIP–ISEs with (**a**) and without (**b**) SWCNTs as a solid contact material.

According to Bobacka’s group [24], chronopotentiometry measurements (CP) of the proposed sensor based MIP were applied in a solution of 9.1 × 10^−4^ M NaDC prepared in 30 mM NaHCO_3_/Na_2_CO_3_ buffer of pH 9.2 at room temperature 25 ± 1 °C. The electrode under study was connected as the working electrode in the presence and absence of SWCNTs. The working electrode was combined with the auxiliary electrode (a glassy carbon (GC) rod) and the reference electrode (Ag/AgCl/KCl (3 M)). The applied current was ±1 nA for 60 s for each amplitude. As mentioned in the chronopotentiogram presented by Figure 7, the overall resistance (*R*) of the electrode was calculated and was found to be 0.7 and 1.1 MΩ for ISEs with and without SWCNTS, respectively. The short-term potential drift was estimated to be 125.2 and 25.3 μV/s in absence and presence of SWCNTs, respectively. The capacitance value of the fabricated electrodes in absence and presence of SWCNTs was calculated to be 7.9 ± 0.6 and 39.5 ± 1.3 μF, respectively. This reflects the significant importance of adding SWCNTs layer as a successful ion-to-electron transducer between the sensing membrane and electronic conductor substrate.

### 3.4. Electrochemical Impedance Spectrometry (EIS)

**Figure 8:**
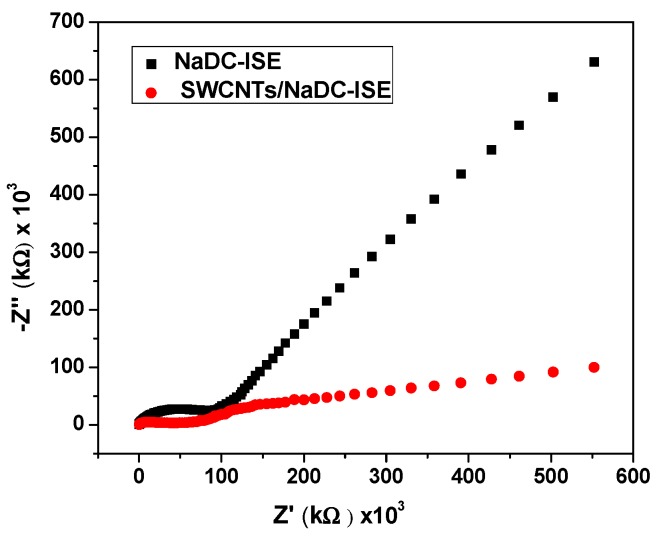
Impedance for NaDC/MIP–ISEs with and without SWCNTs as a solid contact material.

Electrochemical impedance spectroscopy (EIS) was introduced for the proposed ISEs in absence and presence of SWCNTs. The impedance spectra were recorded at open-circuit potential in 0.01 V NaDC solutions with excitation amplitude of 10 mV through a frequency range that starts from 100 kHz to 0.1 Hz. The Nyquist plots were shown in Figure 8. From the high-frequency semicircle, the bulk impedance of the membrane (*R_b_*) can be estimated. The values of (*R_b_*) were found to be *R_b_*= 0.11 and 0.05 MΩ for the proposed ISEs in the absence and the presence of SWCNTs, respectively. The low-frequency capacitance (*C_L_*) for the sensors in absence and presence of SWCNTs were *C_L_* = 2.5 ± 0.9 µF and *C_L_* = 16.2 ± 1.3 µF, respectively. These results confirm the nano-structured features of SWCNTs generate a large double-layer capacitance that revealed an enhanced potential stability of the proposed sensors. All the results arising from EIS and CP measurements confirm that high conductivity and enhanced potential stability of the sensors in presence of SWCNTs.

### 3.5. Selectivity Studies

Studying the interfering ions in the matrix is the most crucial character to exhibit the selectivity efficiency of the proposed sensor in NaDC monitoring. Modified separate solution method (MSSM), which reported by Bakker et al. [25] is represented as the applied method for selectivity studies. The results of selectivity coefficient values in its logarithmic formula were represented in Table 2. The selectivity order of the proposed sensor was in the order: NaDC > Oxalate > Urate ~ PO_4_^3−^ >CH_3_COO^−^ > Cl^−^ > Cholesterol > SO_4_^−2^ ~ NO_3_^−^ > Glucose > Creatinine. The third group (SO_4_^−2^, NO_3_^−^, and glucose and creatinine) show the lower interference and confirm the applicability of the proposed sensor in the presence of these interfering ions. From the obtained results, we confirm the broad suitability of developed sensor for high selectivity determination of NaDC.

### 3.6. Analytical Applications

**Figure 9:**
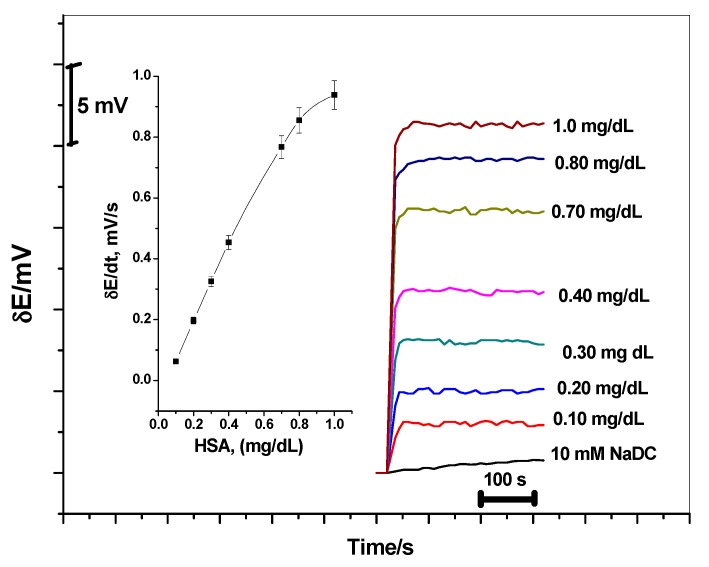
The dynamic potentiometric responses of screen-printed platform towards human serum albumin (HSA) and NaDC at pH = 9.2. The inset shows the measuring calibration plot for HSA.

Serum albumin is major component in plasma and found as human serum albumin (HSA) and bovine serum albumin (BSA), but it is more difficult to determine its concentration. In this report, HSA was easily and rapidly measured potentiometrically. HSA binds with NaDC stoichiometrically as mentioned before [26]. NaDC/MIP–ISE was inserted in 10mL of 10^−2^ M NaDC adjusted to pH 9.2, and different aliquots from 100 mg/dL HSA were added to the solution. The potential of the electrode was recorded for each solution against time. Linear dependence of the initial reaction rate (Δ*E*/Δ*t*) on the HSA concentration was observed with 0.13–0.92 g/dL (Figure 9). At higher HSA concentrations, a slight curvature was noticed. The results showed a relative deviation (RSD %) of ±1.1%. The results obtained for the potentiometric assay of HSA in different serum samples of some different volunteers from different genders at different ages (Table 3). The data agree fairly well with results obtained using the reference method [27,28].

## 4. Conclusions

The presented work showed a full detail for a potentiometric method used for the determination of sodium deoxycholate as a bile acid salt. The presented potentiometric sensors were based on NaDC imprinted polymer (MIP) as sensory element. They were fabricated using coated screen-printed platform and the sensing membrane was modified by single-walled carbon nanotubes (SWCNTs) as an ion-to-electron transducer. The sensors exhibited reliable, acceptable and efficient results for the determination of NaDC in its matrix. They offered long time stability, low potential drift, simplicity in use, ease of miniaturization that ensures high reproducible measurements. Regarding the interaction of NaDC with serum albumin (SA), albumin is determined in human serum samples collected from different volunteers of different ages and gender.

## Figures and Tables

**Table 1:** Potentiometric characteristics of the proposed sensors.

Sensor No.	MIP, mg	DOP, mg	Aliquat, mg	PVC, mg	Slope, mV/decade	Detection Limit, M	Correlation Coefficient, r^2^
1	-	102	4	58	−32.6 ± 0.7	3.5 × 10^−3^	0.997
2	5	102	-	58	−41.6 ± 1.5	3.1 × 10^−4^	0.996
3	5	102	4	58	−47.1 ± 1.2	2.5 × 10^−4^	0.999
4	10	102	4	58	−48.8 ± 2.1	2.0 × 10^−4^	0.999
5	20	102	4	58	−50.3 ± 1.4	7.5 × 10^−5^	0.997
6	30	102	4	58	−60.1 ± 0.9	4.7 × 10^−5^	0.999
7	35	102	4	58	−51.2 ± 0.1	1.0 × 10^−4^	0.998

**Table 2:** Potentiometric selectivity coefficients, Log K^pot^**_x,y_** of the proposed screen-printed ISEs.

Interfering Ion	* Log K^pot^_x,y_ NaDC/MIP-ISE
Cl^−^	−2.5 ± 0.7
SO_4_^−2^	−3.2 ± 0.8
NO_3_^−^	−3.2 ± 0.3
PO_4_^3−^	−1.9 ± 0.7
Glucose	−3.5 ± 0.2
CH_3_COO^−^	−2.2 ± 0.1
C_2_O_4_^2−^	−1.7 ± 0.6
Cholesterol	−2.7 ± 0.3
Creatinine	−3.6 ± 0.4
Urate	−1.9 ± 0.5

* Mean of three measurements.

**Table 3:** Application of the proposed sensor to the determination of albumin in the human blood serum.

Gender	Age	Human Serum Albumin (HSA) g/dL *		Bias %
		Proposed Method ISE	Reference Method [27,28]	
**Male**	1–15	3.5	3.9	9.5
	15–20	4.1	4.5	8.9
	25–40	3.7	4.1	9.8
	45–60	3.1	3.4	8.8
**Female**	1–15	2.6	2.8	8.2
	15–20	3.7	4.1	10.0
	25–40	3.7	4.2	10.0
	45–60	3.4	3.7	8.9

* Average of six measurements.
